# Challenges of Psychiatry Drug Development and the Role of Human Pharmacology Models in Early Development—A Drug Developer's Perspective

**DOI:** 10.3389/fpsyt.2020.562660

**Published:** 2021-01-27

**Authors:** Tong Zhu

**Affiliations:** Astellas Pharma Global Development, Northbrook, IL, United States

**Keywords:** challenges of psychiatry drug development, role of human pharmacology models, three pillars of survival, cognitive impairment associated with schizophrenia, a drug developer's perspective

## Abstract

Psychiatric diseases have the lowest probability of success in clinical drug development. This presents not only an issue to address the unmet medical needs of patients, but also a hurdle for pharmaceutical and biotech industry to continue R&D in this disease area. Fundamental pharmacokinetic and pharmacodynamic principles provide an understanding of the drug exposure, target binding and pharmacological activity at the target site of action for a new drug candidate. Collectively, these principles determine the likelihood of testing the mechanism of action and enhancing the likelihood of candidate survival in Phase 2 clinical development, therefore, they are termed as the “three pillars of survival.” Human Phase 1 pharmacokinetic and pharmacodynamic studies provide evidence of the three pillars. Electroencephalogram (EEG) assessments and cognitive function tests in schizophrenia patients can provide proof of pharmacology and ensure that a pharmacological active regimen will be tested in Phase 2 proof of concept (POC) studies for the treatment of cognitive impairment associated with schizophrenia (CIAS).

## Introduction

Psychiatric diseases have huge unmet medical needs. Schizophrenia is a chronically debilitating syndrome that affects ~1% of the global population and is accompanied by extraordinarily high medical and economic burden (1). Cognitive impairment is one of the three primary clinical symptom domains of schizophrenia (2). Cognitive impairment associated with schizophrenia (CIAS) includes significant deficits [at least 1 Standard Deviation (SD) below performance of healthy control subjects] in attention/vigilance, processing speed, working memory, reasoning, problem solving, verbal memory, visual memory, and social cognition that are present both in patients on antipsychotics as well as drug-naïve patients (3, 4). These impairments have been shown to be associated with negative functional outcomes (5, 6).

Despite the huge unmet needs to treat cognitive impairment as a core feature of schizophrenia, no effective drugs for treating CIAS have been approved. The author of this article presents a perspective on the underlying causes of the low probability of success in clinical development of CIAS treatment, and how human pharmacology models used during phase 1 clinical development can support decision making and mitigate risk for later stage clinical development of CIAS treatment.

## Challenges in Clinical Development of Drugs to Treat CIAS

### Continuing Decline in Pharmaceutical Research and Development (R&D) Productivity

Despite the significant advances in many of the scientific, technological, and managerial factors over the past 60 years, pharmaceutical R&D efficiency, measured simply in terms of the number of new drugs brought to market by the global biotechnology and pharmaceutical industries per billion US dollars of R&D spending, has declined steadily (7). The number of new drugs introduced per year has been broadly flat over the period from 1950s to 2010s and costs have grown steadily. As a result, the pharmaceutical R&D efficiency halves roughly every 9 years over the past 60 years in inflation-adjusted terms (7). There had been many proposed solutions to the problem of declining R&D efficiency, unfortunately, all with limited success.

Multiple factors cause the declining pharmaceutical R&D productivity including the ever-increasing evidential hurdles for product approval, adoption and reimbursement, the progressive lowering of the risk tolerance of drug regulatory agencies, and the tendency for pharmaceutical companies to add resources and complexity to the R&D process. One of the major causes may be related to the shift in the basic research approach for target identification and the high throughput screening methods for lead optimization. Decades ago pharmaceutical research was dominated by low-throughput activities such as animal-based screens and iterative medicinal chemistry. In contrast, research since 1990s utilizes modern molecular biology, genomics-based target identification, automated, high-throughput screening methods for lead optimization. The modern approach does result in molecules of high binding affinity often to a single selected target and of good absorption, distribution, metabolism, and excretion (ADME) characteristics (8). However, the causal link between single targets and disease states is often weaker than thought and biological systems can show a high degree of redundancy, which blunt the efficacy of highly targeted drugs (9, 10). For psychiatric diseases, it is difficult to quantify the engagement of complex neural networks. In addition, targets that are parts of complex networks can lead to unpredictable effects and high affinity molecules especially small molecules can have off-target binding, which lead to toxicity (10, 11). As a result, the probability for small molecules to successfully reach the market has remained rather flat for 60 years, and the overall R&D efficiency has declined due to rising costs (7).

### Low Probability of Success in Psychiatry Drug Development

An analysis of Clinical Development Success Rates was performed by BIO, BioMedTracker and Amplion (12). The analysis dataset included 9,985 clinical and regulatory phase transitions between January 1, 2006 and December 31, 2015 (i.e., Phase 1 to Phase 2, Phase 2 to Phase 3, Phase 3 to New Drug Application approval), which occurred in 7,455 clinical drug development programs across 1,103 companies. The probability of success in each development phase was estimated, and the overall Likelihood of Approval was calculated using the following formula

Likelihood of Approval (LOA) = probability of success in Phase I × Phase II × Phase III × NDA/BLA approval

The analysis breaks down the probability of success by phase and the overall LOA by 14 major disease areas. Each disease area had at least 107 transitions. Psychiatry was one of the major disease areas.

**Figure 1:**
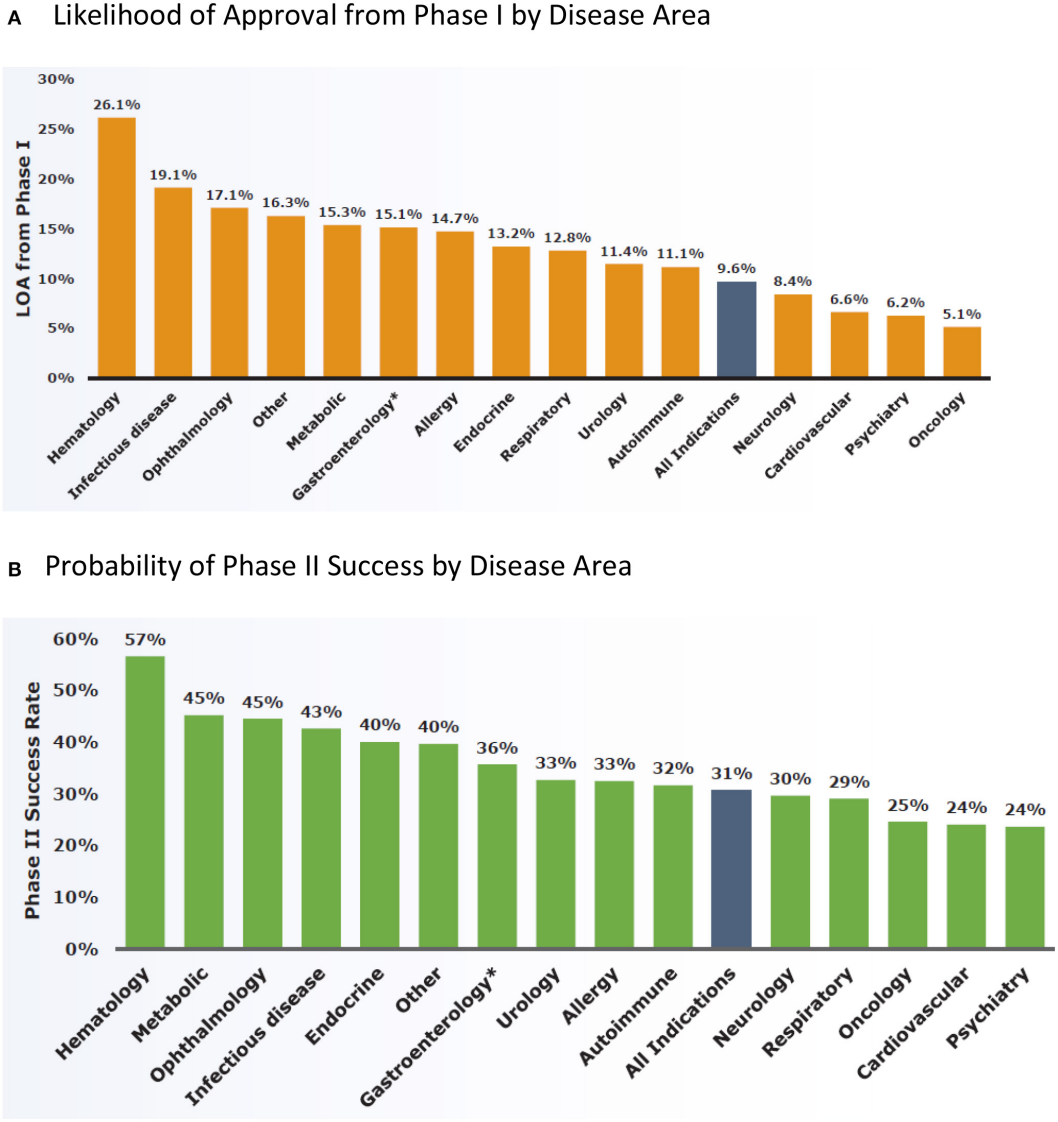
Clinical development success rates between January 1, 2006 and December 31, 2015 analyzed by BIO, BioMedTracker and Amplion (12). **(A)** Likelihood of approval from phase I by disease area. **(B)** Probability of phase II success by disease area.

The overall LOA for all development candidates was 9.6%. Phase 2 is the 'killing ground' for new drugs. The success rate was the lowest in Phase 2 of the four development phases, with only 30.7% of development candidates advancing to Phase 3. The lowest success rate being in Phase 2 was consistent across disease areas. Psychiatry (*n* = 169 transitions) had the lowest overall LOA in non-oncology diseases, 6.2%, as well as the lowest Phase 2 success rate, 24% among the 14 major disease areas (Figure 1) (12).

### Underlying Issues That Cause Failures in Clinical Development of CIAS Treatment

Causes of the low probability of success for psychiatry drug development are likely multi-factorial.

- First is the complex and poorly understood etiology and pathophysiology of the schizophrenia disease. It continues to complicate the identification of proper drug targets. Drug candidate molecules that are designed with high affinity to single targets may have limited efficacy in the complex neural networks or may have off-target effects that restrict their use.
- Second is the limited translational value of animal models used in drug discovery research. Transgenic mice or chemically induced cognitively impaired mice and rats are typical animal models for schizophrenia and CIAS. Neuroanatomical differences between rodents and humans or even other higher animals, and limited behavioral capabilities of rodents lead to increasing questions about the translational value of these animal models. Over-reliance of rodent behavioral models for drug discovery research is thought to be one of the issues leading to lack of efficacy in clinical trials for new drug candidates.
- Third is the clinical trial design features. Clinical trial designs are not only critical to the success of advancing drug candidates in development, but also to determining the approved product labels and ultimately product use in treating the disease. A publication (13) provided a critical review of CIAS trial design and methodology. The authors concluded that underpowering to detect moderate effect sizes, too short of treatment duration (≤8 weeks) and enrolling participants with chronic stable schizophrenia contribute to the failures in CIAS trials. In a recent systemic review (14), among 87 randomized, double-blind, placebo-controlled, add-on pharmacotherapy trials in CIAS patients, only 10 trials (11.5%) required the presence of an objectively assessed cognitive deficit as part of their patient eligibility criteria, and no studies reported stratifying patients according to the presence or degree of cognitive impairment for enrollment. These results suggest that the vast majority of CIAS trials may have been underpowered due to the inclusion of cognitively “normal” patients. A healthy degree of plasticity (i.e., room to move) retained in the cognition-relevant circuitry in schizophrenia patient brains is essential to the success of treatments for CIAS. Because schizophrenia is heterogeneous in presentation and presumably in its underlying pathophysiology, it is very likely that the amount of retained meaningful plasticity differ greatly across patients, and across brain circuitries that are impacted by their diseases (15). Prospectively identifying retained plasticity in patients' cognition-relevant neural circuitries based on objective laboratory measures may be highly beneficial to identifying treatment-sensitive patient populations (15).
- Last but not the least, medication non-adherence is a major problem hampering treatment outcome in schizophrenia patients. Estimated non-adherence rates in schizophrenia are about 50%, ranging widely from 4% (observed in a study with depot neuroleptic drugs) to 72% (16). Adherence varies during the patient's course of illness; it is usually good after hospital discharge and tends to decrease with time. Several prospective studies in schizophrenia patients showed similar results that about one-third of patients were non-adherents after 6 months, and ~50% abandoned the treatment during a year (16). Key drivers of medication non-adherence in schizophrenia include lack of insight, medication beliefs, substance abuse, side effects of medications, and relapse of positive symptoms (17–19).

## Role of Human Pharmacology Models in CIAS Drug Development

Different strategies need to be implemented to address each of the root causes of the low probability of success in psychiatry drug development. Human pharmacokinetic and pharmacodynamic studies can provide evidence that a new drug is achieving adequate exposure at the site of action, fully engaging the pharmacological target and better yet exerting desired pharmacology activities, which enhances the confidence that the molecule will provide clinical benefits to patients. When properly validated, human Phase 1 pharmacokinetic and pharmacodynamic studies are powerful tools to support decision making for further development of new drug candidates.

### Fundamental Pharmacokinetic and Pharmacological Principles Toward Improving Phase 2 Survival

Fundamental principles for a molecule to be effective treating a disease require: (1) achieving free drug exposure at the target site of action at a level that exceeds pharmacological potency over the desired period of time, (2) fully engaging the pharmacological target at the site of action, and (3) eliciting sufficient functional modulation of the target.

A report published by analyzing data from Phase 2 decisions for 44 programs at Pfizer found that not only were the majority of failures caused by lack of efficacy but also that in a large number of cases (43%), it was not possible to conclude whether the mechanism of actions for the molecules had been tested adequately in the Phase 2 trials (20). The analysis revealed positive correlation between the evidence of drug exposure, target binding and pharmacological activity and the program progression or termination. Among the 44 programs, 22.7% (10 out of 44) advanced to Phase 3 whereas in a subset of programs, which had demonstrated drug exposure, target binding and pharmacological activity, the success rate of advancing to Phase 3 was 57.1% (8 out of 14). In contrast, another subset, which had no evidence or only partial evidence of the three aspects, none of the programs advanced to Phase 3 (0 out of 12).

These three fundamental pharmacokinetic and pharmacological principles, i.e., proof of drug exposure, proof of target binding, and proof of pharmacological activity are termed as the “*three Pillars of survival*” (20). They are acknowledged in the PhRMA position paper on best practice for proof of concept (POC) studies (21), as being crucial to “achieve a good POC and reach a definitive answer regarding the utility of potential new therapeutic agents.”

### Human Pharmacokinetic and Pharmacodynamic Studies to Support Decision Making in CIAS Drug Development

For a challenging indication like CIAS, it is essential that we bring biologically active new molecules into clinical POC studies and choose a dose regimen that fully test the mechanism of action of the new molecule in Phase 2 trials. Methodologies to provide evidence of the three pillars in CIAS drug development include

- Proof of drug exposure (Pillar 1): most of the therapeutic targets for CIAS are located in the central nervous system (CNS). Direct measurement of drug concentrations in the human brain is not attainable. Cerebrospinal fluid (CSF) is often used as a surrogate compartment for the brain. Collection of CSF samples can be safely done in Phase 1 pharmacokinetic studies even in healthy volunteers. For molecules that cross the blood-brain barrier via passive diffusion without involvement of active transport mechanism, drug exposure in the blood can be a reasonable surrogate for that in the brain; protein unbound concentrations in the blood should be in equilibrium with concentrations in the brain. Non-clinical pharmacology studies in animals, in which the molecule is administered at various dose levels and drug concentrations and pharmacodynamic effects are measured, usually serve as the basis to determine the target drug exposure in humans. For example, analysis of pharmacokinetic data and pharmacodynamic effects in animals will determine a minimal time-averaged concentration (C_ave_) or a trough concentration (C_trough_) over a dosing interval that is associated with a significant pharmacodynamic effect, as well as the C_ave_ or C_trough_ value associated with the plateau of the pharmacodynamic effect. The determined C_ave_ or C_trough_ becomes the target drug exposure in clinical studies with adjustment of species differences in protein binding and/or binding potency to the target. It is also important to be cognizant about the translatability of animal models to human disease and not to over interpret the value of achieving a target exposure set by animal experiments in predicting clinical success.
- Proof of target binding (Pillar 2): for CNS targets, direct evidence of target binding is most probably obtained from *in vivo* occupancy measurements using positron emission tomography (PET) or radiolabeled ligands (22) in clinical pharmacokinetic and pharmacodynamic studies. Some confidence may be derived in an indirect manner if the binding properties and potency against the target are well-understood (including potential impact of species differences, polymorphisms, or other target phenotypes) combined with a high degree of confidence that adequate target exposure is being achieved (Pillar 1).
- Proof of pharmacological activity (Pillar 3): cognitive function tests and electroencephalogram (EEG) assessments such as auditory steady-state stimulation (ASSR), mismatch negativity (MMN), N100, P200, P300-P3b can be used to demonstrate functional activities for drug candidates in Phase 1 pharmacodynamic studies in schizophrenia patients. Selection of the specific domains in cognitive function tests and the EEG endpoints are determined based on the mechanism of actions of the drug candidate (23, 24). These pharmacodynamic studies are most often placebo-controlled with short treatment durations such as 10–14 days. For the studies to be useful informing decision making for further development of the drug candidate, it is important to establish a minimal effect size on the selected pharmacodynamic endpoint a priori, which would constitute a positive effect of the drug candidate compared to placebo. The minimal effect size should be determined taking into consideration the deficits in schizophrenia patients vs. healthy normal population, clinically meaningful improvement in the endpoints, and assay variability in the measurements. The chosen minimal effect size also influences trial sample size; the study should have adequate power to detect the minimal effect size in the endpoint.
  Functional magnetic resonance imaging (fMRI) is another established neuropharmacological functional marker for CIAS. The fMRI provides a high resolution, non-invasive methodology that enables repeated measures of brain regions activated by stimuli as well as images to assess the intercorrelations among brain regions in response to stimuli. The fMRI is often used in conjunction of cognitive and affective paradigms, which help elucidate the brain systems underlying the behavioral deficits in schizophrenia. For example, by enrolling both CIAS patients and healthy volunteer as controls in studies, contrast images using fMRI revealed reduced activation in regions involved in target and novelty processing in patients accompanied by increased activation in circuits related to elaborated stimulus processing in response to a visual oddball stimulus (25). For targets, abnormal activation was noted in regions related to ideational and visual association, and for novels patients overactivated sensory and frontal areas related to visual spatial processing and working memory (25). Abnormal activation of frontotemporal regions has been associated with more complex downstream processes (25). While fMRI can be a powerful tool for proof of pharmacology, the methodology is most often qualitative and lack the quantitation in physiologic units to support quantitative decision makings for a new drug candidate. The cost, time, and the requirement of specialty centers associated with fMRI also present a challenge to implement it in larger scale, multi-center clinical trials.

Another use of Pillar-3 biomarkers, i.e., pharmacodynamic measures of cognition-relevant brain events in response to a pharmacological stimulation can be to determine whether the brain retains a healthy degree of plasticity (i.e., room to move) in cognition-relevant circuitries. An increase in early auditory information processing (EAIP) after a single-dose challenge of memantine, an uncompetitive NMDA receptor antagonist with low-affinity but rapid on- and off blocking the receptor has been reported as such a measure in schizophrenia patients (15).

## Discussion

Psychiatric diseases have the lowest probability of success in clinical drug development. This presents not only an issue to address the unmet medical needs of patients, but also a hurdle for pharmaceutical and biotech industry to continue R&D in this disease area. Despite the huge unmet needs to treat cognitive impairment as a core feature of schizophrenia, no effective drugs for treating CIAS have been approved.

Fundamental pharmacokinetic and pharmacodynamic principles provide an understanding of the drug exposure, target binding, and pharmacological activity at the target site of action for a new drug candidate. Historical data demonstrated that collectively these principles determine the likelihood of testing the mechanism of action and enhancing the likelihood of candidate survival in Phase 2 clinical development, therefore, they are dubbed as the “three pillars of survival.” For a challenging disease like CIAS, it is essential that we bring biologically active new molecules into clinical POC studies and choose a dose regimen that fully test the mechanism of actions in Phase 2 trials. Human Phase 1 pharmacokinetic and pharmacodynamic studies provide evidence of the three pillars. Cognitive function tests and electroencephalogram (EEG) assessments in schizophrenia patients are invaluable tools for proof of pharmacology for CIAS. For the studies to be useful to support decision making, it is important to establish a minimal effect size on the pharmacodynamic endpoint a priori, which would constitute a positive effect of drug candidate compared to placebo.

One distinction to make is that proof of pharmacology is not necessarily prediction of clinical efficacy. The pharmacodynamic endpoints used in Phase 1 studies such as psychomotor function, attention, working memory and executive function in the cognitive battery test, or ASSR, MMN, N100, P200, P300-P3b in EEG assessments are functional measures. However, they are not necessarily correlated or predictive of the ultimate, composite clinical endpoint used in Phase 2 CIAS trials such as the Measurement and Treatment Research to Improve Cognition in Schizophrenia (MATRICS) Consensus Cognitive Battery (MCCB). Whether or not a POC study will succeed also depends on how the pharmacological target of the drug candidate is manifested in the complex disease pathophysiology, the choice of POC patient population, statistical power of the study, and medication adherence of trial participants. Nevertheless, Phase 1 human pharmacokinetic and pharmacodynamic studies can provide evidence of the three pillars and ensure that a pharmacological active regimen will be tested in the POC study. Pharmacodynamic measures of cognition-relevant brain events in response to a pharmacological stimulation such as a gain in EAIP after an acute memantine challenge may also be used to identify schizophrenia patients who have retained a healthy degree of plasticity (i.e., room to move) in their brain circuitries, which is likely essential for an intervention to success in CIAS (15).

## Data Availability Statement

All datasets presented in this study are included in the article/supplementary material.

## Author Contributions

TZ conceptualized the perspective presented in this article, analyzed the data, wrote the manuscript, and approved the finale submitted version of the manuscript.

## Conflict of Interest

TZ is an employee of Astellas Pharma Global Development, USA.
